# Opportunities and challenges for anti-CD47 antibodies in hematological malignancies

**DOI:** 10.3389/fimmu.2024.1348852

**Published:** 2024-02-23

**Authors:** Yilan Xu, Panruo Jiang, Zhenyan Xu, Haige Ye

**Affiliations:** ^1^Department of Hematology, The First Affiliated Hospital of Wenzhou Medical University, Wenzhou, China; ^2^Department of Hematology, Dongyang People’s Hospital, Jinhua, China

**Keywords:** cd47, anti-CD47 antibody, immunotherapy, hematological malignancies, magrolimab

## Abstract

CD47 is a cell-surface ligand that is overexpressed in various malignancies and that binds to SIRPα on macrophages to promote tumor cell evasion of phagocytosis. Blocking the CD47-SIRPα axis can increase the phagocytosis of macrophages to exert antitumor effects. CD47-based immunotherapy is a current research focus. The combination of anti-CD47 antibodies with other drugs has shown encouraging response rates in patients with hematological tumors, but side effects also occur. Bispecific antibodies and SIRPα/Fc fusion proteins appear to balance the efficacy and safety of treatment. We review the latest clinical research advances and discuss the opportunities and challenges associated with CD47-based immunotherapy for hematological malignancies.

## 1 Introduction

Hematological malignancies are malignant tumors originating from the lymphatic and hematopoietic systems and are characterized by high malignancy, complex treatment, and poor prognosis. The combination of multiple chemotherapeutic drugs is a classic treatment for patients with hematological malignancies (1). However, due to the strong heterogeneity of molecular characteristics, many patients still suffer relapse and resistance without personalized and precise treatment (2–5). In recent years, tremendous advances in immunotherapy have been observed (6–8). These approaches targeting the adaptive immune system have been widely used for the treatment of various hematological malignancies to improve patient prognosis. In addition, the innate immune system, which serves as the first line of defense against the external environment, plays an important role in cancer cell surveillance and elimination (9, 10). Therapies targeting the innate immune system may offer additional hope for the treatment of hematological malignancies.

**Figure 1:**
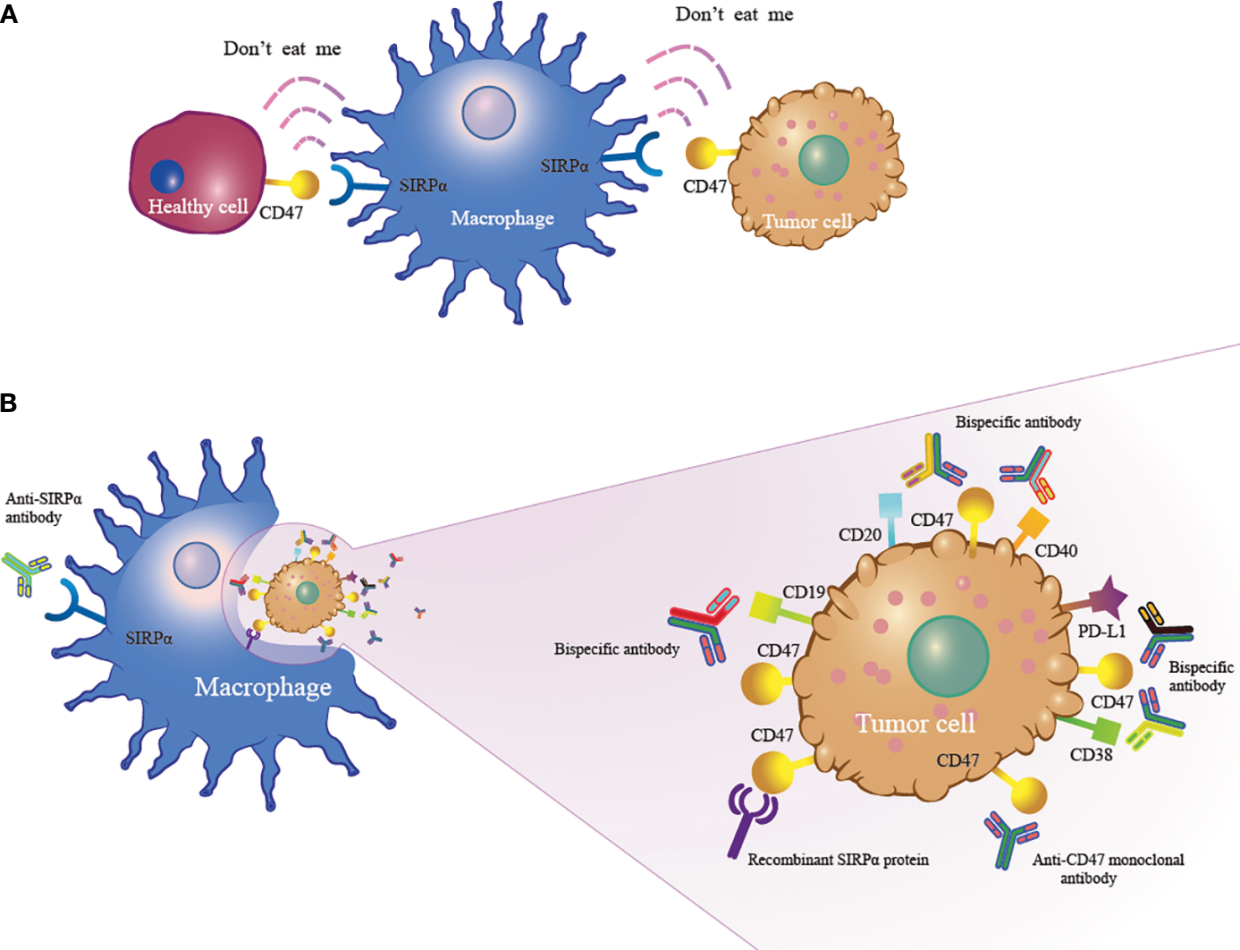
The mechanism of CD47-based immunotherapy. **(A)** CD47 expressed on tumor cells binds to SIRPα on macrophages to activate the “don’t eat me” signal to enable tumor cells to escape macrophage-mediated phagocytosis. **(B)** Anti-CD47 monoclonal antibody, anti-SIRPα antibody, recombinant SIRPα protein and bispecific antibody inhibit the CD47-SIRPα interaction, leading to macrophage phagocytosis of tumor cells.

CD47, recognized as an innate immune checkpoint protein, is a cell surface ligand overexpressed in various hematological and solid tumor malignancies (11–14). CD47 binds to signal-regulating protein alpha (SIRPα) on macrophages to trigger the “don’t eat me” signal that protects cancer cells from macrophage-mediated phagocytosis (**Figure 1A**). Blocking the CD47-SIRPα axis can increase the phagocytosis of macrophages to exert antitumor effects (**Figure 1B**) (15–17). Currently, inhibitors targeting the CD47-SIRPα axis are being developed worldwide, and they are in preclinical and clinical study phases. The combination of anti-CD47 antibodies and other drugs has shown encouraging response rates in patients with hematological tumors, but side effects also occur. Bispecific antibodies and SIRPα/Fc fusion proteins appear to balance the efficacy and safety of treatment. In this article, we review the new developments in CD47-based immunotherapy for hematological malignancies. In addition, we discuss the potential and challenges of targeting the CD47-SIRPα axis in the treatment of hematological malignancies.

## 2 CD47 monoclonal antibody

### 2.1 Magrolimab

Magrolimab, a humanized monoclonal antibody against CD47, is currently being evaluated in several clinical trials for hematological malignancies. A phase 1 trial of magrolimab with azacitidine had meaningful efficacy, with an overall response rate (ORR) of 75% and a complete remission (CR) rate of 33% in patients with higher-risk myelodysplastic syndrome (MDS) (18), while the ORR and CR rates were much lower with the single agent azacitidine in pivotal trials (ORR< 60%, CR rates< 20%) (19, 20). Encouraging results were also observed in TP53-mutated patients receiving combined treatment comprising magrolimab and azacitidine, and the CR and marrow complete remission (mCR) rates of TP53-mutated MDS patients were 38% and 13%, respectively. The CR rate of TP53-mutated acute myeloid leukemia (AML) patients was 59% (21). These results are in good agreement with those of previous clinical trials in which TP53-mutated MDS and AML patients were treated with azacitidine, for which the CR rate was < 22% (22, 23). Magrolimab was shown to decrease the frequency of TP53 mutation alleles in this clinical trial, which led to improved drug response rates. Triple therapy with magrolimab, azacitidine and venetoclax was evaluated in 74 AML patients. This triple combination has an ORR of 75% in patients with relapsed or refractory (R/R) AML previously not treated with venetoclax, with greater responses (ORR 80%) in newly diagnosed (ND) AML patients. However, for patients who were previously exposed to venetoclax, the ORR was only 12%. In this study, 24% of patients (18/74) experienced ≥ Grade 3 anemia, but no anemia-related life-threatening events or deaths occurred (24). However, Gilead Sciences announced the discontinuation of the phase 3 enhance study of magrolimab plus azacitidine in patients with higher-risk MDS in July 2023 suddenly for undisclosed reasons. Gilead Sciences announced that its phase 3 enhance study should be stopped in AML patients with TP53 mutations. Compared with standard of care, magrolimab is unlikely to demonstrate a survival benefit in patients with AML harboring TP53 mutations.

The promising efficacy of magrolimab plus rituximab was shown in R/R non-Hodgkin lymphoma (NHL) patients. Among 15 patients with diffuse large B-cell lymphoma (DLBCL), the ORR and CR rate were 40% and 33%, respectively. Among the 7 patients with follicular lymphoma, the ORR and CR rate were 71% and 43%, respectively. The median duration of response (DOR) was not reached at a median follow-up of 6.2 months and 8.1 months (25). In addition, the combination of magrolimab, rituximab, gemcitabine and oxaliplatin produced encouraging results in R/R DLBCL patients, with an ORR of 51.5% and a CR rate of 39.4% (26). After a median follow-up of 11.3 months, the median DOR was 18 months, and the median overall survival (OS) was not reached. Similar results were observed in a historical study of 196 patients with R/R DLBCL treated with rituximab plus gemcitabine and oxaliplatin (R-GemOx), for which the ORR was 54%. With a median follow-up of 22 months, the median OS was 10 months. However, the CR rate for these patients was only 23% (27). The poor ORR of R/R DLBCL patients receiving combination therapy comprising magrolimab, rituximab and acalabrutinib was 28%, and the study was stopped early due to the lack of significant clinical synergy between the three drugs (28). In addition to tumor cells, erythrocytes also highly express CD47 (29, 30), which leads to accelerated clearance of erythrocytes in patients treated with magrolimab, resulting in severe hemolytic anemia. However, these adverse events were mitigated by administering a lower priming dose of magrolimab (31). Many possibilities have been demonstrated for the use of magrolimab in the treatment of hematological malignancies, and multiple combinations of magrolimab and other drugs are currently undergoing clinical trials (32, 33).

### 2.2 Letaplimab (IBI188)

Letaplimab is another traditional humanized anti-CD47 monoclonal antibody that has certain antitumor effects but inevitably leads to anemia. For 45 evaluable patients treated with letaplimab and azacitidine in a phase 1b trial, 82.2% of patients achieved an ORR, with 31.1% achieving CR. The incidence of anemia among these patients was 48% (34).

### 2.3 Lemzoparlimab

Lemzoparlimab is an anti-CD47 antibody screened using human-derived natural bacteriophage technology that can specifically target tumor cells to circumvent hematological adverse events by reducing binding to erythrocytes. Lemzoparlimab is now being tested in ND higher-risk MDS patients in a phase 2a trial (35). Among 28 evaluable patients who received ≥ 3 cycles of treatment with lemzoparlimab and azacitidine, the ORR was 82.1%.

### 2.4 Ligufalimab (AK117)

Ligufalimab is now being investigated in ND AML patients in a phase 1b trial (36). A total of 40 patients were enrolled and received combination therapy comprising ligufalimab and azacitidine. The most frequently reported Grade ≥ 3 adverse events (AEs) were leukopenia (52.5%), thrombocytopenia (47.5%), neutropenia (45.0%), lymphopenia (25.0%), and anemia (25.0%). Among the 20 evaluable patients, 9 patients achieved CR, and 2 achieved complete remission with incomplete hematological recovery (CRi). After a median follow-up of 6.7 months, the median DOR was not reached. Another phase 1b study conducted from the ligufalimab study conducted in ND high-risk MDS patients showed that among 27 evaluable patients, the CR rate was 48.1%. AK117 was also well tolerated and was associated with a low incidence of anemia in MDS patients, and 22.2% of patients experienced Grade ≥ 3 anemia (37).

### 2.5 Maplirpacept (PF-07901801)

Maplirpacept is currently being tested in a phase 1b/2 study in patients with R/R DLBCL (38). In phase 1b, researchers will determine the maximum tolerable dose of maplirpacept and determine the doses of tafasitamab and lenalidomide. In phase 2, researchers will explore the objective response of patients receiving this triple combination treatment.

### 2.6 AUR103

AUR103 is an oral small molecule inhibitor of CD47 and is currently in a phase 1 trial (39). There are currently no publicly available data.

## 3 SIRPα/Fc fusion protein

### 3.1 Evorpacept (ALX148)

Evorpacept is a high-affinity CD47-blocking fusion protein with an inactive human immunoglobulin Fc region. It can promote macrophage phagocytosis of tumor cells but has almost no effect on normal blood cells. The results from phase 1a in ND and R/R AML showed that evorpacept in combination with venetoclax and azacitidine was safe and tolerable (40). However, in August 2023, ALX Oncology announced the termination of the recombinant protein ALX148 in MDS and AML due to poor efficacy. This may be because ALX148 engineered with an inactive Fc effector has fewer side effects but attenuates the effect on tumor cells.

### 3.2 IMM01

IMM01, a recombinant human SIRPα fusion protein, can bind to CD47 on the tumor cell membrane to mediate macrophage phagocytosis of tumor cells. Preclinical data revealed that IMM01 has the unique characteristic of weak human erythrocyte conjugates that prevent severe hemolytic anemia.

The preliminary results of a phase 1 trial showed that IMM01 monotherapy was well tolerated in R/R lymphoma patients, with only four patients (13.8%) experiencing anemia (Grade ≥3) (41). In addition, a phase 2 trial of IMM01 with azacitidine demonstrated its efficacy, with an ORR of 88.2% and a CR rate of 41.2% in patients with ND higher risk MDS. With a median follow-up of 5.6 months, the median DOR was not reached (42). Encouraging results were also observed in ND chronic myelomonocytic leukemia (CMML) patients receiving combined treatment comprising IMM01 and azacitidine; the ORR was 88.9%, and the CR rate was 44.4%. The CR rate increases with increasing treatment time, but the median DOR was not reached (43).

Furthermore, IMM01 can present tumor antigens to T cells through MHC molecules to exert dual antitumor effects. The combination of IMM01 and tislelizumab (an anti-PD-L1 antibody) had synergistic effects on more patients with classic Hodgkin lymphoma (cHL), with an ORR of 64.3% and a disease control rate (DCR) of 100%. IMM01 demonstrated good tolerability and safety among these patients, with no reported hemolytic anemia or hemolysis (44).

### 3.3 BYON4228

BYON4228 is a novel humanized SIRPα antibody with high specificity for SIRPα that maximizes the antitumor immune response and silences the Fc backbone to reduce toxic effects. BYON4228 is currently in a phase 1 trial to evaluate its safety and efficacy in R/R B-cell NHL (45). No clinical data on BYON4228 have been reported thus far.

## 4 Bispecific antibody

### 4.1 IBI322

IBI322, an anti-CD47/PD-L1 bispecific antibody, is highly selective for tumor cells but mitigates the effects of other cells. It enables macrophages to phagocytose lymphoma cells and promotes antitumor cytotoxic T-cell immune responses. A phase 1 study revealed that IBI322 monotherapy was safe and effective for anti-PD-1or PD-L1 treatment-resistant cHL patients. Among the 23 evaluable patients, the ORR and DCR were 47.8% and 91.3%, respectively. Lymphopenia is the most common AE (Grade ≥3) and occurs in approximately 29.2% of patients, while no patients experienced AE-induced discontinuation or death (46).

### 4.2 TG-1801

TG-1801 is a bispecific antibody designed with one arm blocking CD47 and the other arm binding to CD19 to accurately identify tumor cells. A combination treatment of TG-1801and ublituximab (an anti-CD20 antibody) was evaluated in 16 R/R B-cell lymphoma patients (47). The ORR was 44%, with one patient achieving CR and 6 patients achieving partial response (PR).

### 4.3 IMM0306

IMM0306 is a bispecific antibody that targets CD47 and CD20, and a higher affinity for CD20 results in a better binding preference to malignant B cells and more effective anti-lymphoma activity. IMM0306 monotherapy therapy is currently in a phase 1/2 trial to evaluate its safety and efficacy in R/R B-cell NHL (48).

Moreover, many CD47-targeted bispecific antibodies that are engineered to specifically target other surface proteins on tumor cells while blocking the CD47-SIRP axis can produce synergistic antitumor effects. These agents are currently in phase 1 trials to evaluate their safety and effectiveness (49–51).

## 5 Discussion

The CD47-SIRPα axis is a novel antitumor target that has shown promising results in clinical studies for the treatment of hematological malignancies. However, as related studies have progressed, questions about CD47-based immunotherapy have emerged, such as about the limited efficacy of single agent therapy and biosafety issues.

Compared with those designed on a human IgG1 scaffold, anti-CD47 antibiotics engineered on a human IgG4 scaffold can minimize the Fc-dependent effector functions of innate immunity, such as antibody-dependent cell-mediated cytotoxicity (ADCC) and antibody-dependent cellular phagocytosis (ADCP) (52, 53). CD47 is widely expressed in normal cells, so many companies have chosen to develop human IgG4-type antibiotics to reduce damage to normal cells, which weakens the antitumor effect of these anti-CD47 antibody monotherapies (53, 54). Therefore, it is necessary to combine an anti-CD47 antibody with other drugs to enhance antitumor activity. The selectivity of anti-CD47 antibodies for tumors depends not only on blocking antiphagocytic signals but also on the extensive expression of prophagocytic signals. Azacitidine is a cytotoxic agent that induces the endogenous expression of cell surface calreticulin in AML and MDS cell lines. Cell surface calreticulin serves as an identified prophagocytic signal that binds to its macrophage receptor, low-density lipoprotein-related protein, resulting in phagocytosis of target cells (55, 56). The combination of azacitidine and magolizumab not only blocks the “don’t eat me” signal but also activates the “eat me” signal, resulting in significantly greater macrophage-mediated cellular phagocytosis of cells than that of cells treated with either drug alone. In addition to cytotoxic agents, other drugs that can induce cell apoptosis, such as the combination of magrolimab, venetoclax and azacitidine, also have synergistic effects on cells treated with anti-CD47 antibodies. Moreover, Dr. Boasman reported that the combination of a Jak inhibitor (ruxolitinib) and an anti-CD47 antibody increased the expression of calreticulin, signaling a much stronger prophagocytic message in cells derived from primary myelofibrosis patients (57). The tumor microenvironment is complex, and in addition to calreticulin, abnormal expression of the regulatory protein macrophage inhibitory factor (MIF) can also have a great impact on the survival of tumor cells (58). MIF, which is associated with most cancers in all stages, can modify the activation, adherence, and phagocytosis of macrophages. In addition to its immunological functions, MIF is considered to play a role in cell proliferation and differentiation. Dr. Li studied the tumor microenvironment of multiple myeloma (MM) patients and reported that one of the significant changes was the reprogramming of macrophages, which results in phagocytic dysfunction (59). An MIF inhibitor can correct this effect. A dual-macrophage-targeted therapeutic strategy involving the combination of an MIF inhibitor and an anti-CD47 antibody activated phagocytosis and repolarized macrophages to a functional phenotype and demonstrated potent antitumor effects *in vitro* and *in vivo*. In addition, anti-CD47 antibody-mediated phagocytosis can be enhanced by combination with tumor-targeting antibodies. The anti-CD20 antibody rituximab exerts effects by binding to Fc receptors on natural killer (NK) cells (16). The Fc domain of rituximab provides a potent prophagocytic signal for macrophages by stimulating ADCP. In rituximab-resistant patients, the combination of magrolimab plus rituximab improves antitumor activity through blockade of the antiphagocytic CD47 signaling pathway combined with rituximab-mediated activation of ADCP via the Fc domain (25). Although CD47 has been recognized as an innate immune checkpoint, studies have shown that blockade of the CD47-SIRPα axis can increase the cross-presentation of antigens, leading to adaptive antitumor immune responses initiated and activated by T cells. Thus, T-cell responses could be enhanced by the combination of T-cell checkpoint inhibitors (anti-PD-1 and PD-L1 antibodies) and anti-CD47 antibodies (60). A clinical trial has been initiated to evaluate drugs targeting the CD47-SIRα axis and tislelizumab (an anti-PD-L1 antibody) in lymphoma patients (44).

Specifically, erythrocytes are a significant exception to normal cells, as they express prophagocytic signals in certain environments. Moreover, erythrocytes also highly express CD47, which is involved in the protection against erythrocyte clearance. After receiving anti-CD47 antibodies, senescent erythrocytes acquire CD47 blockade in the presence of enhanced prophagocytic signals, leading to accelerated clearance and ultimately to anemia. This adverse event was mitigated by administering a lower priming dose of magrolimab, which eliminated older erythrocytes and preserved younger erythrocytes lacking prophagocytic signals. Although this procedure still resulted in transient mild anemia, the patient’s anemia was relieved to some extent with a compensatory increase in reticulocytes. Moreover, erythrocytes exposed to the priming dose rapidly shed CD47 from the cell surface through a process called erythrocyte pruning, shielding erythrocytes from the effects of subsequent doses of magrolimab (61, 62). Furthermore, increasing the selectivity of antibodies for tumor cells is an option for reducing anemia. Lemzoparlimab, a novel anti-CD47 antibody, did not cause severe anemia to develop when it mediated the phagocytosis of tumor cells. This is due to glycosylation near the binding epitope on erythrocyte CD47, which “protects” erythrocytes from lemzoparlimab binding. In addition, 82.1% of MDS patients treated with lemzoparlimab achieved an ORR without serious anemia (35). In addition to modifying anti-CD47 antibodies to target a distinct CD47 epitope, recombinant SIRPα can also reduce hematological adverse events. Among these recombinant proteins, ALX 148 and IMM01 are the most promising and are currently being evaluated in clinical studies of hematological malignancies. However, ALX Oncology announced the termination of the recombinant protein ALX148 in MDS and AML due to poor efficacy. Although an inactive Fc effector reduces biosafety issues, it also limits the effectiveness of treatment. In addition, SIRPα is highly expressed on central and peripheral nervous system cells, and some anti-SIRPα antibodies may lead to the loss or dysfunction of nerve cells, resulting in neurological dysfunction (63, 64).

How to avoid accidental injury to normal cells while exerting antitumor effects is the most important problem that needs to be considered in the development of CD47-based immunotherapy in the future. In this situation, many bispecific antibodies have emerged; one arm blocks CD47, while the other arm binds to common cancer antibody targets. In addition, an “imbalanced” design with a decreased binding affinity to CD47 and increased binding affinity to tumor cell surface proteins can retain tumor-specific phagocytic stimulation activity while retaining host cells to limit toxicity. It is crucial to identify and select surface biomarkers for hematological malignancies. In addition, novel drug delivery carriers based on nanoparticles are also good choices because of their small molecular weight, precise targeting, and easy modification (65). Multifunctionalized iron oxide magnetic nanoparticles, which are carriers of anti-CD47 antibodies, not only help to retain their targeting activity but also achieve a short-term increase in delivery to cancer cells, accelerating cancer cell apoptosis (66). Nanoparticles loaded with an anti-CD47 antibody achieved antitumor effects in a 4T1 tumor-bearing mouse model by continuously releasing the antibody to block the CD47-SIRPα axis (67). In addition, identifying the optimal timing and approach for introducing drugs may also lead to greater efficacy and toxicity reduction (68–70). All of these questions require further exploration.

In conclusion, the CD47-SIRPα axis is a promising antitumor target, and multiple CD47-targeted drugs have entered clinical trials. The latest clinical research advances are listed in **Table 1**. Although there are difficulties in the development of CD47-based immunotherapy for hematological malignancies, such as poor efficacy and hematological side effects, these issues may be solved by the development of bispecific antibodies and the establishment of new drug delivery systems. However, further results are worthy of our anticipation.

**Table 1:** Clinical trials of CD47-based immunotherapy.

Drug	Code	Phase	Target	Combination	Indication	Age range (years)	Population Size(n)	Grade≥3 AEs	Results		Reference
Magrolimab	NCT03248479	1	CD47	Azacitidine	ND and R/R MDS	NA	56	NA	TP53-mutated MDS:CR:38%, mCR:13% TP53 wild-type MDS:CR:30%, mCR:35%		(21)
					ND and R/R AML	NA	23	NA	TP53-mutated AML: CR:59%, CRi or CRh:9% TP53 wild-type AML:CR:46%		
Magrolimab	NCT04435691	1b/2	CD47	Venetoclax +Azacitidine	ND and R/R AML	NA	74	Febrile neutropenia (50%), pneumonia (38%), anemia (24%), bilirubin elevation (11%), transaminitis (11%), creatinine elevation (8%), hypokalemia (8%)	R/R AML ORR	VEN exposed: 12% VEN-naïve: 75%	(24)
									ND AML ORR: 80%		
Magrolimab	NCT02953509	1b	CD47	Rituximab +Gemcitabine+Oxaliplatin	R/R DLBCL	31-86	33	Anemia (60.6%), thrombocytopenia (42.4%), neutropenia (18.2%)	ORR: 51.5%, CR: 39.4%, median DOR: 18 months, median OS not reached,		(26)
Magrolimab	NCT03527147	1	CD47	Acalabrutinib +Rituximab	R/R DLBCL	51-84	7	Anemia (42.9%), thrombocytopenia (28.6%), bilirubin elevation (14.3%), vomiting (14.3%), transaminitis (14.3%)	Stopped		(28)
Magrolimab	NCT05835011	2	CD47	Decitabine/Cedazuridine	ND higher risk MDS	NA	Recruiting	NA	In progress		(71)
Magrolimab	NCT04086264	1b/2	CD47	Pivekimab sunirine	R/R AML	NA	Recruiting	NA	In progress		(72)
Magrolimab	NCT04892446	2	CD47	Daratumumab/Pomalidomide+Dexamethasone/Carfilzomib+Dexamethasone	R/R MM	46-82	25	NA	Safe and well-tolerated		(32)
Magrolimab	NCT04599634	1	CD47	Venetoclax+Obinutuzumab	R/R indolent B-cell malignancies	NA	Recruiting	NA	In progress		(33)
IBI188	NCT04485065	1b	CD47	Azacitidine	ND higher risk MDS	NA	93	NA	ORR: 82.2%, CR:31.1%, mCR:35.6%		(34)
Lemzoparlimab	NCT04202003	2a	CD47	Azacitidine	ND higher risk MDS	NA	53	NA	ORR: 82.1%		(35)
AK117	NCT04980885	1b	CD47	Azacitidine	ND AML	NA	40	leukopenia (52.5%), thrombocytopenia (47.5%), neutropenia (45.0%), lymphopenia (25.0%), anemia (25.0%)	CR:45%, CRi:10%; median DOR not reached		(36)
AK117	NCT04900350	1b	CD47	Azacitidine	higher risk MDS	NA	72	Anemia (22.2%)	CR:54.2%		(37)
Maplirpacept	NCT05626322	1b/2	CD47	Tafasitamab +Lenalidomide	R/R DLBCL	NA	Recruiting	NA	In progress		(38)
AUR103	NCT05607199	1	CD47	/	AML/MDS	NA	Recruiting	NA	In progress		(39)
Evorpacept (ALX148)	NCT04755244	1a	CD47	Venetoclax +Azacitidine	ND and R/R AML	50-82	14	Febrile neutropenia(43%), anemia (43%), transaminitis (36%), thrombocytopenia (29%), pneumonia (21%)	Safe and well-tolerated		(40)
IMM01	CTR20191531	1	CD47	/	R/R lymphoma	19-75	29	Anemia (13.8%), thrombocytopenia (10.3%), leukopenia (6.9%).	Safe and well-tolerated		(41)
IMM01	NCT05140811	2	CD47	Azacitidine	ND higher risk MDS	30-83	54	Leukopenia (81.5%), thrombocytopenia (68.5%), neutropenia (66.7%), lymphopenia (57.4%), anemia (44.4%), infection(16.7%)	ORR: 88.2%, CR:41.2%, median DOR not reached		(42)
					ND CMML	NA	24	Lymphopenia (66.7%), neutropenia (54.2%), leukopenia (54.2%), thrombocytopenia (54.2%), anemia (20.8%), pneumonia (16.7%)	ORR: 88.9%, CR:44.4%, median DOR not reached		(43)
IMM01	NCT05833984	2	CD47	Tislelizumab	anti-PD-1cHL	23-73	20	Lymphopenia (30.0%), thrombocytopenia (10.0%), neutropenia (5.0%), leukopenia (5.0%)	ORR:64.3%, DCR:100%		(44)
BYON4228	NCT05737628	1	SIRPα/Fc fusion protein	Rituximab	R/R CD20+ B-NHL	NA	Recruiting	NA	In progress		(45)
IBI322	NCT04795128	1	PD-L1,CD47	/	anti-PD-1cHL	25-68	24	Lymphopenia (29.2%)	ORR:47.8%, DCR:91.3%		(46)
TG-1801	NCT03804996	1	CD19,CD47	Ublituximab	R/R B-cell lymphoma	NA	16	NA	ORR: 44%		(47)
IMM0306	CTR20192612	1/2	CD20,CD47	/	R/R CD20+ B-NHL	N/A	26	Lymphopenia (65.4%), leukopenia (23.1%), neutropenia (15.4%), anemia (11.5%), thrombocytopenia (7.7%)	Safe and well-tolerated		(48)
ISB 1442	NCT05427812	1/2	CD38,CD47	/	R/R MM	NA	Recruiting	NA	In progress		(49)
CPO107	NCT04853329	1/2	CD20,CD47	/	R/R CD20+ B-NHL	NA	Recruiting	NA	In progress		(50)
SL-172154	NCT05275439	1	CD40,CD47	Azacitidine	R/R AML and higher risk MDS	NA	Recruiting	NA	In progress		(51)

AEs, adverse events; ND, newly diagnosed; R/R, relapsed or refractory; MDS, myelodysplastic syndrome; CR, complete remission; mCR, marrow complete remission; AML, acute myeloid leukemia; CRi, complete remission with incomplete hematological recovery; CRh, complete remission with partial hematological recovery; ORR, overall response rates; VEN, venetoclax; DLBCL, diffuse large B-cell lymphoma; DOR, duration of response; OS, overall survival; CMML, chronic myelomonocytic leukemia; cHL, classical Hodgkin lymphoma; DCR, disease control rate; B-NHL, B-cell non-hodgkin lymphoma; MM, multiple myeloma.

## Author contributions

YX: Writing – original draft. PJ: Writing – original draft. ZX: Writing – original draft. HY: Writing – review & editing.
